# Cultural factors associated with the intent to be screened for prostate cancer among adult men in a rural Kenyan community

**DOI:** 10.1186/s12889-017-4897-0

**Published:** 2017-11-23

**Authors:** Kinyao Mutua, Anne M. Pertet, Careena Otieno

**Affiliations:** 1 Kenya Medical Training Institute, P.O Box 260, Sultan Hamud, Kenya; 2 Kenya Medical Training College, Machakos, Kenya; 3 Great Lakes University of Kisumu (Gluk), P. O. Box: 2224-40100, Kisumu, Kenya

**Keywords:** Prostate cancer, Screening, Intention, Beliefs, Family influence, Fatalism, Fear, Benefits, Demographic characteristics

## Abstract

**Background:**

The aim of this study was to determine cultural factors associated with prostate cancer screening intent among adult Kenyan African men.

**Methods:**

A cross-sectional quantitative study with an analytic design was carried out in a randomly selected sample of 155 adult men aged 25–98 years living in a rural community in Kenya. Constructs from the Theory of Planned Behaviour were used to guide this study. A 5 -point Likert scale was used to assess fatalistic beliefs, fear, perceived benefits, and family influence. A structured questionnaire was used to collect quantitative data at the household level.

**Results:**

Only 2.4% of the study participants had been screened for prostate cancer. About 2/3rd (64%) of the participants felt that they were at risk of getting prostate cancer; 44% intended to be screened within the following 6 months. Mean scores on a 5-point Likert scale indicated: strong beliefs in the benefits of prostate screening (4.2 (±SD .8), men aged over 40 were not perceived to be at risk of getting prostate cancer (1.3 ± .6), relatively high fatalistic beliefs of prostate cancer screening (3.6 (±SD .8), high degree of fear or apprehension of prostate cancer screening (3.2 (±SD 1.2), and a high level of influence of family members in prostate cancer screening (3.9 (±SD 1.0). The Wald criterion demonstrated that only family influence made a significant contribution to the intent to screen for prostate cancer (*p* = 0.031). Age, education, marital status, fatalism, fear, and benefit of screening were not associated with the intent to screen for prostate cancer.

**Conclusions:**

Strong beliefs of the benefits of prostate screening tended to be surpassed by relatively high fatalistic beliefs and fear or apprehension in prostate cancer screening. The family plays an important role in influencing decision making related to prostate cancer screening in Africans.

## Background

For reasons that remain unclear, men of African origin have the highest rate of incidence for prostate cancer (PCa) in the world [1]. For example, the incidence of prostate cancer in African Americans is almost 60% higher, and the mortality rate is two- to three-times greater than that of Caucasian American men [2]. A recent systematic review on PCa in Africa which provided a continent-wide incidence rate of PCa, estimated an overall pooled incidence of 21.95/100,000 population. This incidence rate tended to increase by age group. For example, the incidence rates were: 39.0/100,000 population in people aged 70 years and above; 25.0/100,000 population in men 60–69 years; 16.3 and 12.9 per 100,000 population in 50–59 years and 40–49 years respectively [3]. In Kenya, Age Standardised Rate (ASR) is 40.6/100,000. Prostate cancer accounts for 17.3% of all male cancers and 10.2% of all the other cancers in Kenya [4].

In Africa, excessive mortality rates from prostate cancer are associated with higher mortality when compared to other regions of the world. This pattern is attributed to limited availability of screening and early detection [5]. Available statistics show that only 50% of African American men are screened for prostate cancer [6]. In Africa, these rates are much lower, ranging from 0 to 11% [7, 8]. In Kenya, between 4.1% and 11% of males are screened for prostate cancer [9, 10]. Furthermore, the majority (87.5%) of patients who attend the Kenyan health facilities come when the cancer is at an advanced stage, i.e. stages III(C) and IV(D) [11]. Furthermore, despite massive education campaigns on prostate cancer awareness in Kenya, the screening rate is still low [12]. The question therefore is, why are so few men being screened despite the knowledge? Is this an issue of personal and behavioural factors or is it associated with broader social and contextual factors, such as cultural influence which shape behaviour?

To answer this question, we used the Theory of Planned Behaviour (TPB) [13], a theory that links beliefs and explains human behaviour. The theory states that individual behaviour is motivated by behavioural intentions, which is a function of a person’s attitude and beliefs toward the behaviour, the influence of the individual’s social environment, and the individual’s perceived control over resources and skills necessary to perform the behaviour. This theory provided the framework for testing the relationship between intent to screen and: behavioural beliefs (fatalistic beliefs, fear, benefits of screening), normative beliefs (influence of significant others, and perceived behavioural control beliefs which are all governed by social interactions that are culturally influenced.

Cultural factors related to cancer screening have been examined, in understanding why some groups choose to adopt or not adopt recommended behaviours [14, 15]. These factors include among others attitude and beliefs: fatalistic beliefs (events beyond individuals control); fear of screening; perceived benefits of screening and family influence (relatives, peers, prostate cancer survivors, etc.) [16, 17].

Qualitative studies indicate that African Americans are more likely to embrace fatalistic beliefs and fear, resulting in delayed diagnosis [18, 19]. These findings are supported by quantitative studies that show fear as being positively associated with cancer screening, even after controlling for background variables such as social, economic status and education [20]. A study using the Theory of Planned Behaviour showed that African Americans held relatively weak fatalistic beliefs; a small degree of fear/apprehension and strong beliefs in the benefits of screening [21]. The challenge in research is to determine at what point fear becomes a barrier or a facilitator to screening. Perceived benefit of prostate cancer screening has been associated with intent to screen in quantitative and qualitative studies conducted in African Americans even after controlling for the association of fatalism and fear [20, 21]. Perceived benefits had a statistically significant correlation (*r* = 0.285, *p* = 0.018) with prostate cancer screening intent.

Family pressure may, to some extent, influence decision making and adherence to prostate cancer screening. Qualitative studies conducted to identify significant beliefs, barriers, and motivators associated with prostate cancer screening behaviours revealed a positive influence of significant others (relatives, spouses, peers, prostate cancer survivors) in promoting cancer screening. The family played a major role in influencing decision-making related to prostate cancer screening [17, 21–27]. In an African American study, which used the Theory of Planned Behaviour, social pressure of family members was significantly associated with intent to screen (*r* = 0.337, *p* = 0.005) [26].

In summary, most evidence on prostate cancer screening intent has emerged from qualitative studies among African American and Caucasian American men. There is currently a paucity of quantitative research that explains prostate cancer screening behaviours of African men. Additionally, research using theoretically driven approaches to account for the role of culture in shaping health-related behaviours particularly in Africa is limited. Thus, our study sought to establish cultural factors such as beliefs, attitudes, and family influence related to screening intent in Africans in order to come up with educational interventions to improve prostate cancer screening.

We addressed the following research questions: a) Is fear and fatalism associated with prostate cancer screening intent b) Is perception of the benefits of prostate cancer screening associated with prostate cancer screening intent? c) Does the family influence decisions related to prostate cancer screening intent? The null hypotheses were, a) there is no significant association between, a) behavioural beliefs and b) normative beliefs and perceived behavioural control.

## Methods

### Study population and site

Fisher’s et al. (1998) adapted by Mugenda and Mugenda 2003 [28], n = z^2^pq/d^2^ was used determine the sample size. n = desired sample size; Z = the abbisca of normal distribution (z = 1.96); p = the proportion of the population tested for prostate cancer nationally (11%); q = 1-P (proportion not tested; d = maximum degree of error with a confidence interval of 95% = 0.05. This calculation gave a minimum sample size of 155.

Although cancer of the prostate occurs in men who are 40 years and above, this study purposely included all adult males found in the household who had some knowledge of causes and symptoms of prostate cancer. Only men who answered any one of the questions on knowledge of prostate cancer, or mentioned one symptom of prostate cancer were included in the study. Most of the youth were either in school or away from home. Those found at home during the study period happened to be aged over 25 years. We excluded those who were mentally unsound or unable to communicate.

The study was carried out in Kasikeu County Assembly, Makueni County, Kenya a rural ward. The ward was randomly selected from 30 County Assemblies in the County between October 2014 and February 2015. The population of males in this ward was 4569 while that of females was 4933 making a total population of 9502 according to the 2009 census.

Kasikeu County Assembly comprises of 37 villages consisting of 2047 households. Each village formed a primary sampling unit (PSU), while the households in the village were secondary sampling units (SSUs). The PSUs (villages) did not have the same number of SSUs (households). Thus, we selected the PSUs using Probability Proportional to Size sampling (PPS) which gave large PSUs a greater probability of occurring in the sample than small PSUs. The households were spread across the County Assembly. We moved to the first sampled household, and if the head of household had neither heard of nor knew at least one symptom of prostate cancer we moved to the nearest household until we got the eligible study subject. To get the 155 target household heads we visited 420 households.

### Study design and data sources

We used an analytical, cross-sectional design to examine the relationship between cultural variables related to behavioural beliefs, and normative beliefs with perceived behavioural control. Perceived behavioural control was assessed as the indication of a person’s readiness to screen for prostate cancer within a six-month period. Behavioural beliefs (fatalism, fear, and benefits) and normative beliefs (family influence) were the independent variables, while social demographic characteristics were possible intervening variables.

Questions used in this study were adapted from the Thomas Jefferson University Prostate Cancer Screening tool [29, 30], which drew on health behaviour models (i.e., Health Belief Model, Theory of Reasoned Action, Social Cognitive Theory). The tool was used to assess factors associated with screening frequency among African American men [26].

We used a structured questionnaire to collect quantitative data through face to face interviews. The questions used a forced choice categorical response to obtain consistent information from all the participants.

The participants were asked whether they intended to be screened for cancer in the subsequent 6 months. This question had a 3-point response (i.e. 1 = yes, 2 = no, 3 = don’t know). “Don’t know” response was treated as a ‘No’ response.

The basis for this classification was that “Don’t know” response was equivalent to participants who were uncertain and thus their responses could not be classified as a definite yes. Treating “Don’t know” as a yes would have biased the proportion of those who were sure that they intended to be screened. Beliefs and family influence items were measured using a 5-point Likert (i.e. 1 = strongly disagree, 2 = disagree, 3 = neither agree nor disagree, 4 = agree, 5 = strongly agree). Item total scores were used to derive two categories for beliefs and family influence.

Items related to fatalism included: “*If I am meant to get prostate cancer I will get it no matter what I do,” and “If I have prostate cancer I would just as well not know about it.”*

Items related to fear/apprehension included: “*I am bothered by the possibility that prostate cancer screening might be physically uncomfortable,” and “I am afraid that if I have a prostate screening test, the results will show that I have it.”.*

Items related to perceived benefits included: *“I think the benefits of prostate cancer screening outweigh any difficulty I might have in going through the tests,” and “I believe that prostate screening is an effective way to treat prostate cancer early.”*

Family influence was assessed as the perceived social pressure of a family member.

Items included in family influence were: *“I want to do what members of my family think I should do about prostate cancer screening,” and “Members of my family are likely to suggest I should go through prostate screening.”*

One item measured screening history. The question was whether one had received a PSA test. This question had a 3-point response (i.e. 1 = yes, 2 = no, 3 = don’t know). “Don’t know” responses for PSA were treated as a ‘No’ response. The premise behind this re-categorization was that a “no” response was equivalent to participants who did not know whether or not they received a PSA test, or were not informed or given the results of the test.

Data on social demographic characteristics which included age, education, religion and marital status were collected as possible modifying variables as well as for describing the sample.

Before data collection, research assistants were trained to ensure standardization of procedures and integrity of the data. Specific practices included: a review of procedures for recruitment of the sample, training on the data collection tool, interviewing techniques, seeking consent, maintaining confidentiality, and survey administration.

After receiving the consent to participate in the study, the research assistants explained the study’s aims, the interview process, and the approximate length of time it would take to complete the interview. The respondents were also given an opportunity to ask questions on the study before being interviewed. Interviews lasted between 15 to 25 min.

### Reliability and validity

The questionnaire was pre-tested before the survey started. Questions similar to our study had been tested in a similar study conducted among African Americans [27]. In their study, content validity was established with five cancer health professionals who provided suggestions for the questionnaire. Using factor analysis, they found a reliability of 0.61. Construct validity based on factor analysis and factor loading was 0.35 or more. Cronbach’s α for the fear/apprehension scale was 0.67. The internal consistency using a Cronbach’s α was 0.77.

On the other hand, in our study, we established content validity with three health social scientists who provided suggestions on the appropriateness of the questions and the Likert scale items. We pre-tested the items selected from the Thomas Jefferson University Prostate Cancer Screening Survey on 15 men to establish whether these questions reliably measured the intention to be screened. The internal consistency of the scores using values of Cronbach’s α for the four subscales were 0.69, 0.67, 0.71, and 0.78, for fatalism, fear/apprehension, perceived benefits and family influence respectively. Widely-accepted cut-off for items considered in social science research is an alpha of .70 or higher. However, a cut-off of 0.60 is common in exploratory research [31].

### Data management and analysis

Data were entered using Statistical Package for the Social Sciences (SPSS), version 20 for Windows data file which had a participant’s unique identification number. Data checking and cleaning methods included examining ranges of responses for each individual variable through frequency distributions. SPSS was used to evaluate missing data for possible oversight upon entry, normality, and outliers. Missing data were addressed through list wise deletion which excluded variables that had missing data. The advantage of this approach is that the process produced true relationship matrices. Using this procedure, we found that there were no cases excluded from analysis due to missing data in our study.

Fisher test of skewness was used to assess whether or not the continuous data were normally distributed.

Frequency distributions and percentages were used to describe the social demographic characteristics of the study participants and to summarize key study variables. Counts and proportions were used to summarize the categorical variables such as marital status, educational level, or categorized Likert scale data. The Likert scale data was treated as a continuous variable with an interval scale. Total subscale scores were created for fatalism, fear/apprehension, perceived benefits, and family influence before statistical inferences were made. Means and standard deviations were used to summarize continuous variables. Furthermore, Likert scale scores of the independent variables were categorized into two classifications using the 50th percentile of the frequency distributions.

An analytical, cross-sectional design helped to establish the associations between cultural variables related to beliefs, normative beliefs, perceived benefits, and the intent to be screened for prostate cancer. Chi Square statistic was used to test the association between categorical independent variables (social demographic characteristics, fatalism, fear/apprehension, benefits and family influence) with the intent to screen, which was the dependent variable. The null hypotheses were that there were no significant relationships between any of the independent variables and the dependent variable. Decisions for statistical significance of the findings were made using an alpha level of <0.05. Binary logistic regression analysis was used to determine which of the independent variables best explained or the intention to screen.

All variables added to the logistic regression model had significant relationships as determined through the Chi square statistic at a *p* value of <0.05. The variables which were found to be significant from the Chi square statistic were selected as a block (using the ‘enter ‘procedure in SPSS) in a single step into the model.

### Ethical considerations

Approval for the study was granted by the Great Lake’s University of Kisumu (GlUK) Institutional Review Board (IRB), Ref. No. GREC/158/27/2014. Research assistants explained to the participants that their involvement was voluntary and that they were free to withdraw from the study at any time, *without giving a reason*. The respondents could also refuse to answer questions they were uncomfortable with, and their data could be removed from the study if they so wished. The study participants were assured of anonymity and confidentiality of the information they provided and were also assured that they would not be identified by name but by an identification number. The consent was mostly oral or thumb printed. No incentives were provided to take part in the study.

## Results

### Social-demographic characteristics of the sample

Detailed social demographic data and prostate cancer screening are given in Table 1. The eligible sample for this study consisted of 155 adult men. The sample mean age was 49.8 ± 16.7 years which ranged from 25 to 98 years. One-third (32.9%) of the participants were less than 40 years. Majority of the respondents (85%) were married and (94%) were Christians. The level of education measured using the Kenyan school system showed that 25% of the men had no formal education, while only 6.4% had tertiary education indicating low levels of education.

**Table 1:** Socio-Demographics and Prostate Cancer-Related Characteristics of the Sample by Intention to Screen *(n = 151)*

Factor	Variable	Percent	P value
Age in years	<40	32.9	0.934
	>40	67.1	
Marital status	Single	15.9	0.302
	Married	84.1	
Education level	No Formal Education	24.6	0.155
Primary Level		75.4	
At Risk of getting Prostate cancer	Yes	63.7	0.079
Screened for prostate cancer	Yes	2.4	

Chi square test was used to assess the association between the various demographic factors and intent to screen. This analysis excluded four men who had been screened for prostate cancer. The results show no significant associations (*p* > 0.05) between social demographic variables of age, marital status, and education on intent to be screened for prostate cancer.

### Prostate cancer screening

We assessed screening history of prostate-specific antigen blood test (PSA) and digital rectal examination (DRE). Only four men out of the 155 (2.4%) had been screened for cancer. When asked whether they perceived themselves as being at risk of developing prostate cancer, 64% of men reported that they felt at risk of developing prostate cancer. However, using the Likert scale, perception that men aged over 40 were a risk group had a weak mean score of 1.3 ± .0.6 showing they did not believe men aged over 40 were a risk group. Forty-four (43.6%) of the men said they intended to be screened within the following 6 months.

### Beliefs on prostate cancer screening

Fatalism had a mean score of 3.6 (±SD 0.8) indicating that this sample held relatively high fatalistic beliefs about prostate cancer and prostate cancer screening. About 2/3rd of the sample held fatalistic views. They believed that if they were meant to get prostate cancer nothing could stop it, and they would rather not know about it.

A fear mean score of 3.2 (±SD 1.2) indicates a relatively high degree of fear or apprehension associated with prostate cancer screening. Thirty-seven percent (37%) of the men were bothered that prostate cancer screening might be physically uncomfortable and were afraid of a positive prostate screening test.

Perceived benefits of screening had a mean score of 4.2 (±SD .8), representing strong beliefs in the benefits of screening. The perception was that benefits of screening outweighed any discomfort of testing, and believed that prostate screening is an effective way to treat prostate cancer early. Close to 90% of the men agreed that the benefits of prostate cancer screening outweighed any difficulty they might have in testing, and that prostate screening was an effective way to treat prostate cancer early.

### Family influence

A family influence mean score of 3.9 (±SD 1.0) represented a high level of influence of family members on prostate cancer screening. The majority (89%) of the men reported that they would do what members of their immediate family thought they should do regarding prostate cancer screening.

### Relationships between attitude and beliefs and family influence with intent to screen

This section establishes the likelihood that the 151 respondents would report intention to be screened for prostate cancer. This sample excluded the four men who had been screened. The outcome variable (intention to screen for prostate cancer in the following 6 months) was coded 1, while the intention *not* to be screened was coded 0. SPSS version 20 logistic regression tool was used to compute a binary logistic regression which was the odds probability of belonging to either group (1/0). A model (i.e. an equation) was created that included variables that were useful in explaining intention to screen for prostate cancer. These variables included age, marital status, fatalism, fear, family influence, perceived benefits. All these variables were entered at the same time providing only one model. Since this model was applied to data where the dependent variable was categorical, the composite variable predicted the probability of a case being in category 1.

The null model, i.e. the model with no explanatory variables indicated that the dependent variable in the equation correctly classified 56.8% of the respondents. When the explanatory variables were added, the correct classification increased to 58.8% indicating only a slight improvement. Omnibus tests of model coefficients which provide information on whether the inclusion of the block of explanatory variables contributed significantly to model fit had a *p* value of 0.06 showing that the model had a significant influence on the null model. When the model fit was evaluated to assess which of the explanatory variables improved the model, only family influence was significant with a *p* value of 0.018.

The goodness of fit which indicates the appropriateness of the model, and how well it fits the actual outcome was estimated with H-L test. Our results had a p value of 0.294 indicating that the model was appropriate (a significant of *p* > 0.05 indicate the model fitted the data well). The statistical significance of individual explanatory variables was then tested using Wald chi square statistic which provided an index of the significance of each of the six explanatory variables individually, while controlling for the other explanatory variables. The Wald criterion demonstrated that only family influence made a significant contribution (*p* = 0.031) to the intent to screen for prostate cancer. Age, marital status, fatalism, fear, benefit did not significantly contribute to the screenig intent.

Measures of log likelihood which are tentative indicators of the range of which the actual influence of the explanatory variables on the dependent variable lay, showed that the explanatory variables explained only 3 to 4% of the variation in the dependent variable using Cox & Snell R^2^ and Nagelkerke R^2^ respectively.

## Discussion

The aim of this study was to determine cultural factors associated with prostate cancer screening intent among adult Kenyan African men using an analytical, cross-sectional design.

Examining cultural constructs and social variables in the context of culture was needed to understand why some groups choose to adopt or not adopt recommended behaviours. Although guidelines recommend that adult men be screened at 40 years and above, we purposely included the younger age group as these cultural factors might affect their decisions to be screened at the recommended time.

The Theory of Planned Behaviour which links beliefs and behavior and which explains human behavior was considered appropriate for the study. This theory provided a framework for the examination behavioral beliefs and normative beliefs. Behavioral beliefs included fear/apprehension, fatalistic beliefs, and perception of the benefits of screening. Normative beliefs are individual’s perception of prostate cancer screening, which are influenced by the judgment of significant others (e.g., parents, spouse, friends, physician).

Our findings suggest that this population perceived themselves to be at risk of getting prostate cancer, but did not believe it was men aged over 40 who were most at risk. Though they were aware of the benefits of screening, very few had been tested. The population held high levels of fatalistic beliefs that they would get cancer no matter what they did, and were also fearful that screening would be painful and tests could be positive. However, these factors (fatalistic beliefs and fear and benefits of testing) were not associated with prostate cancer screening intent. Our findings suggest that prostate cancer screening intent was best explained by pressure from the family or family influence.

The influence of the family on behaviour in this context is consistent with previous quantitative and qualitative studies which have highlighted familial influence in decision-making related to prostate cancer screening among African-American men [19, 21–27]. These findings indicate that the influence of the family in prostate cancer screening was evident in African men likewise.

We discuss the rest of our results and make comparisons with a study of African Americans which used the Theory of Planned Behaviour and Thomas Jefferson University Prostate Cancer Screening tool [26]. Our results on fatalism and fear were inconsistent with those found in African Americans. We found that whereas African men held relatively high fatalistic beliefs and relatively elevated levels of fear/apprehension, African Americans had weak fatalistic beliefs and showed a small degree of fear. These differences in fear and fatalism could be due to the different cultural backgrounds. Furthermore, African Americans might have been more enlightened on prostate cancer, compared to our population which was less well educated and probably less informed. The two studies reported that the benefits of prostate cancer screening outweighed perceived barriers to screening. However, contrary to our study where we found no relationship between perceived benefit and screening, the American study found a significant association between perceived benefit of testing and prostate cancer screening intent.

The above findings are consistent with the wealth of qualitative literature that has consistently indicated beliefs as relevant variables in prostate cancer screening [19, 20]. The concept of perceived benefits as an important factor in prostate cancer screening behaviours has also been reported in other studies [22, 23].

### Limitation of the study

Though our sample size was based on a formal power calculation, which provided a sample size representative of this sub culture, the results cannot be generalized to a larger population. Furthermore, studies on culture cannot be easily generalized due to diversities in cultures. Nonetheless, this sample was much larger than the one used in the African American population described earlier which used the Theory of Planned Behavior. In the latter study, a minimum sample size of 80 was arrived at using guidelines from Cohen [31] with an analysis of power for multiple regression at 0.78 level of power with *p* < 0.05, and assuming a mean correlation coefficient of *r* = 0.4 to detect a 16% shared variance between six predictor variables of the theory of planned behavior [26]. Despite the limitations of sample size, the study came up with several findings with respect to prostate cancer-related beliefs, family influence and prostate cancer screening intent which can be followed up in studies using larger sample sizes.

Explanatory variables explained only 3 to 4% of the variation in prostate cancer screening intent. This calls for inclusion of other variables from the theory of planned behaviour which were not addressed in our study. These include cost, time, embarrassment, and discomfort, including situational barriers to screening and fear related barriers such as fear of diagnosis, treatment, fear of sexual dysfunction. Social factors which include social, economic status (SES) such as income and education and insurance coverage, could also delay prostate cancer screening. Thus, there is need to conduct more studies to explain the link between SES and prostate cancer screening. Finally, our study did not include qualitative aspects which could have explained the quantitative study findings.

### Strengths of our study

Evidence shows a scarcity of research that explains prostate cancer screening behaviours of African men, as most studies have focused on African American men. This study thus contributes further to the body of knowledge on cultural aspects of prostate cancer screening by extending a similar study to Africans. Furthermore, although the Theory of Planned Behaviour has been well supported by empirical evidence in African American men, its constructs have not been extensively applied in examining prostate cancer screening behaviours of African men. This study, therefore, contributes to the body of research in this aspect by providing an interpretive framework on the interrelationships using the Theory of Planned Behaviour.

## Conclusions

Our study suggests that while behavioural beliefs are important in prostate cancer screening intent, normative beliefs (family influence) made the most significant contribution to prostate cancer screening intent in this sub-culture. Low prevalence of prostate cancer screening intent could be attributed to fear and fatalistic beliefs. These findings underscore the importance of targeting fear and apprehension including further reinforcement of the family role in public health interventions. Further research which includes more constructs of the Theory of Planned Behaviour in different African setups is recommended. This would help develop interventions designed to meet the diverse cultural aspects of particular populations. Pilot interventions using family influence approaches that could improve screening of prostate cancer in Kenya are recommended.

## Abbreviations

ASR: Age standardised rate
DRE: Digital rectal examination
GlUK: Great Lake’s University of Kisumu
IRB: Institutional Review Board
PCa: Prostate cancer
PPS: Probability proportional to size
PSA: Prostate-specific antigen
PSUs: Primary sampling unit
SSUs: Secondary sampling units
TPB: Theory of planned behaviour
